# Adipose tissue fibrosis in human cancer cachexia: the role of TGFβ pathway

**DOI:** 10.1186/s12885-017-3178-8

**Published:** 2017-03-14

**Authors:** Michele Joana Alves, Raquel Galvão Figuerêdo, Flavia Figueiredo Azevedo, Diego Alexandre Cavallaro, Nelson Inácio Pinto Neto, Joanna Darck Carola Lima, Emidio Matos-Neto, Katrin Radloff, Daniela Mendes Riccardi, Rodolfo Gonzalez Camargo, Paulo Sérgio Martins De Alcântara, José Pinhata Otoch, Miguel Luiz Batista Junior, Marília Seelaender

**Affiliations:** 1 Cancer Metabolism Research Group, Institute of Biomedical Sciences, University of Sao Paulo, Sao Paulo, Brazil; 2 Department of Surgery, Faculty of Medicine, University of Sao Paulo, Sao Paulo, Brazil; 3 Department of Clinical Surgery, Hospital University, University of Sao Paulo, Sao Paulo, Brazil; 4 Biotechnology Group, Laboratory of Adipose Tissue Biology, University of Mogi das Cruzes, Mogi das Cruzes, Brazil; 5 Department of Nutrition, Federal University of Sao Paulo, Sao Paulo, Brazil; 6 Faculty of Nursing, University of Campinas, Campinas, Brazil

**Keywords:** Cancer cachexia, Fibrosis, Adipose tissue, Extracellular matrix, TGFβ

## Abstract

**Background:**

Cancer cachexia is a multifactorial syndrome that dramatically decreases survival. Loss of white adipose tissue (WAT) is one of the key characteristics of cachexia. WAT wasting is paralleled by microarchitectural remodeling in cachectic cancer patients. Fibrosis results from uncontrolled ECM synthesis, a process in which, transforming growth factor-beta (TGFβ) plays a pivotal role. So far, the mechanisms involved in adipose tissue (AT) re-arrangement, and the role of TGFβ in inducing AT remodeling in weight-losing cancer patients are poorly understood. This study examined the modulation of ECM components mediated by TGFβ pathway in fibrotic AT obtained from cachectic gastrointestinal cancer patients.

**Methods:**

After signing the informed consent form, patients were enrolled into the following groups: cancer cachexia (CC, *n* = 21), weight-stable cancer (WSC, *n* = 17), and control (*n* = 21). The total amount of collagen and elastic fibers in the subcutaneous AT was assessed by histological analysis and by immunohistochemistry. TGFβ isoforms expression was analyzed by Multiplex assay and by immunohistochemistry. Alpha-smooth muscle actin (αSMA), fibroblast-specific protein (FSP1), Smad3 and 4 were quantified by qPCR and/or by immunohistochemistry. Interleukin (IL) 2, IL5, IL8, IL13 and IL17 content, cytokines known to be associated with fibrosis, was measured by Multiplex assay.

**Results:**

There was an accumulation of collagen and elastic fibers in the AT of CC, as compared with WSC and controls. Collagens type I, III, VI, and fibronectin expression was enhanced in the tissue of CC, compared with both WSC and control. The pronounced expression of αSMA in the surrounding of adipocytes, and the increased mRNA content for FSP1 (20-fold) indicate the presence of activated myofibroblasts; particularly in CC. TGFβ1 and TGFβ3 levels were up-regulated by cachexia in AT, as well in the isolated adipocytes. Smad3 and Smad4 labeling was found to be more evident in the fibrotic areas of CC adipose tissue.

**Conclusions:**

Cancer cachexia promotes the development of AT fibrosis, in association with altered TGFβ signaling, compromising AT organization and function.

## Background

Cancer cachexia is an irreversible syndrome in which involuntary weight loss occurs, due to skeletal muscle mass and adipose tissue wasting. This condition is associated with poor prognosis, and decreased survival [1, 2]. Cachexia is seldom diagnosed or treated, despite affecting around 80% of all cancer patients [3], and being the direct cause of 22–40% of cancer deaths [4, 5]. The typical metabolic dysregulation acts in concert with the increase of inflammatory mediators [6, 7], as the syndrome develops in the manner of a chronic inflammatory state.

Profound wasting of WAT is frequently observed during cachexia, and recent evidence suggests that it precedes protein breakdown in the skeletal muscle or any decrease in food intake [8, 9]. The WAT has been recognized as a highly dynamic organ [10–12]. Therefore, changes in WAT may be proposed as early markers of the syndrome, thus presenting a potentially valuable tool for precocious diagnosis. Previous studies [9, 13–16] have shown that the AT is deeply affected by local inflammation during cancer cachexia. Several pro-inflammatory factors, such as TNFα, IL-1β and IL-6 are up-regulated by in the adipose tissue during cachexia, and we have shown before [14–16], that the subcutaneous depot presents a relevant contribution to systemic inflammation as these factors reach the circulation.

Each particular anatomical depot of adipose tissue exhibits specific arrangement and functions, along with diverse morphology and varying density of adipocytes, of pre-adipocytes, of fibroblasts, of endothelial cells and of resident macrophages [10, 17]. The extracellular matrix (ECM) is a complex network essential for tissue architecture and cell functioning [10, 18]. ECM acts as one of the most important reservoirs of growth factors, metalloproteinases, collagens and fibronectin. Indeed, changes in ECM rapidly trigger signaling pathways controlling different cell functions, including development, migration, proliferation, apoptosis, and gene expression [19]. Among the different extracellular matrix components, collagen VI is highly expressed in the adipose tissue, and is considered as the key form secreted by adipocytes. The lack of collagen VI in col6 KO ob/ob mice results in the uninhibited expansion of individual adipocytes [20, 21]. Studies with mice bearing the cachexia-inducing tumor MAC-16 show that adipocyte dimensions are reduced, the cell membranes disrupted, and that the tissue suffers fibrosis [9].

Fibrosis results from persistent inflammation, in which activated mechanisms for repair response lead to excessive accumulation of extracellular matrix components [22, 23]. TGFβ is one of the key cytokines taking part in wound-healing and tissue fibrosis [24]. Binding of TGFβ to membrane receptors causes the assembly of a receptor complex that, in turn, phosphorylates SMAD proteins [25–27]. Through the action of SMADs on target genes, the induction of differentiation of fibroblasts into myofibroblasts takes place [28]. Myofibroblasts are contractile cells expressing alpha-smooth muscle actin (α-SMA), involved in the production of ECM proteins, such as collagen and fibronectin [29, 30].

Myofibroblast activation, proliferation and survival are also mediated by other pro-inflammatory cytokines: TNF, IL13, IL1 and TGFβ [31–34]. The microenvironment in fibrosis triggered by the TGFβ pathway often leads myofibroblasts to sustain uncontrolled deposition of extracellular matrix components, since these cells become resistant to the mechanisms of apoptosis [23].

Recently, we demonstrated that cachectic patients with gastrointestinal cancer show morphological rearrangement in the subcutaneous AT, resulting in adipocyte size reduction, AT atrophy, formation of fibrotic areas and immune cell infiltration [35]. In the current study, we hypothesized that the morphological changes in cancer cachexia concomitant with augmented expression of ECM elements occurs through enhanced signaling of the TGFβ pathway.

## Methods

### Patient recruitment

All subjects were selected between 2012 and 2015 at the ambulatory unit of the Surgical Medical Clinic at the University Hospital, after being scheduled for exploratory laparotomy or abdominal surgery (*n* = 212). The adopted exclusion criteria were: liver or kidney failure, AIDS, chronic inflammatory processes not related to cachexia, chemotherapy treatment (at the time), and chronic anti-inflammatory therapy. All the procedures were performed according to the Declaration of Helsinki, and were approved by the Ethics Committee of Research Involving Human Subjects of the Institute of Biomedical Sciences/University of Sao Paulo (1082/CEP) and by the Human Ethics Committee of the University Hospital/University of Sao Paulo (CEP 752/07). The fully informed written consent signature was obtained from every patient after a detailed explanation of the study. Following engagement in the study, patients were divided into three groups. The control group (C) included weight stable patients subjected to surgical hernia removal. Patients diagnosed previously with gastrointestinal cancer were divided in two groups: Weight-stable Cancer Group (WSC) and Cachectic Cancer Group (CC). The WSC group was comprised of patients with greater than 5% body weight loss in the previous 6 months. CC group included the patients with gastrointestinal cancer and cachexia, characterized according to the following criteria: weight loss greater than 5% in fewer than 12 months, fatigue, anorexia; and abnormal biochemical parameters (increased inflammatory markers: C-Reactive Protein, anemia, and by low serum albumin) [1]. The questionnaire EORTC QLQ-C30 was applied to assess quality-of-life of all patients and the obtained data indicated a reduced overall quality-of-life in the cachectic group. A total of 153 patients were excluded from the study. Forty patients refused to participate. Further exclusion criteria were inconsistent data in questionnaires (*n* = 19) and BMI greater than 29.9 kg/m^2^ (*n* = 03). Patients of the control group showing inflammation (CRP > 5 mg/L) (*n* = 27) and anemia (*n* = 01) were also excluded. Some patients were excluded due to unconfirmed diagnosis after the final pathological analysis, as well as due to insufficient adipose tissue sample allowing analysis, or which could not be matched with a corresponding blood sample (*n* = 63). A total of 59 patients were recruited into the study. Table 1 shows the characteristics of the study groups.

**Table 1:** General and clinical characteristics of studied groups

	CONTROL	WSC	CC	*P value*
*Clinical Parameters*				
N	21	17	21	
Male/Female (n)	16/5	9/8	13/8	
Height (m)	1.65 ± 0.09	1.62 ± 0.09	1.63 ± 0.1	0.6735
Age (years)	54.10 ± 14.89	62.13 ± 11.78	63.00 ± 11.0	0.0642
Previous body mass (Kg)	71.43 ± 13.32	73.50 ± 13.28	73.78 ± 13.86	0.8431
Current body mass (Kg)	71.43 ± 13.32	69.28 ± 12.19	63.62 ± 12.90	0.1577
Δ Body mass (Kg)^a^	0.00 [0.00; 0.00]	0.00 [−7.2; 0.0]	−8.0 [−12.50;−7.0]	*P* < 0.0001*
BMI (Kg/m^2^)	25.82 ± 3.27	25.79 ± 4.62	23.68 ± 3.49	0.1558
*Biochemical Parameters*				
C-Reactive Protein (mg/dL)	0.11 [0.0; 0.23]	0.1950 [0.0; 0.4]	1.170 [0.72; 1.3]	<0.0001***^#^
Albumin (g/dL)	4.75 [4.2; 5.1]	4.37 [4.05; 4.64]	3.84 [2.79; 4.58]	0.0037**
Hemoglobin (g/dL)	15.25 [13.9; 15.8]	13.10 [11.30;14.08]	11.80 [9.15; 13.20]	<0.0001***^#^
IL6 (pg/ml)^a^	0.00 [0.00; 0.84]	0.62 [0.00;1.77]	4.10 [1.25;9.74]	0.0008***^#^
TNFα (pg/ml)^a^	5.42 [4.16; 6.15]	6.01 [3.92; 7.52]	8.82 [6.08; 13.87]	0.028**
IL2 (pg/ml)^a^	ND	ND	ND	
IL5 (pg/ml)^a^	0.00 [0.00;0.73]	0.00 [0.00;0.58]	0.85 [0.00;1.36]	*p* < 0.0282^#^
IL8 (pg/ml)^a^	0.52 [0.35;2.16]	4.79 [2.02;6.47]	28.91 [17.36;68.86]	*p* < 0.0001*^#^
IL13 (pg/ml)^a^	0.03 [0.03;0.05]	0.04 [0.03;0.04]	0.07 [0.02;0.105]	0.2947
IL17^b^ (pg/ml)^a^	0.79 [0.71;1.28]	1.09 [0.79;1.41]	1.56 [0.87;2.31]	0.1305

ANOVA one way; Data are presented as mean and S.E.M. ^a^Kruskal Wallis Test; Data presented as median, 1st and 3st quartile. To cytokines content: Control (*n* = 17), WSC (*n* = 13), CC (*n* = 12); ^b^Control (*n* = 10), WSC (*n* = 9), CC (*n* = 10). *p < 0.05 **p < 0.003 ***p < 0.0001 CC vs Control; ^#^ *p* < 0.05 CC vs WSC. *TNFα* tumour necrosis factor α; *IL6* interleukin 6; *IL2* interleukin 2; *IL5* interleukin 5; *IL8* interleukin 8; *IL13* interleukin 13; *IL17* interleukin 17

### Adipose tissue samples

Subcutaneous adipose tissue was collected during the surgery procedure. Fat pad slices were rapidly frozen in dry ice and maintained in 4% paraformaldehyde solution (w/v) for the histological analysis described below. For protein and gene expression, analyses of the samples were maintained at -80 °C, prior to processing.

### Plasma and serum measurements

Approximately 20 ml of blood were collected during admission at the hospital, and aliquots of both plasma and serum was stored at -80 °C for later measurements. All analyses were performed in the automatic LABMAX 240® equipment from Labtest, using commercial standards for Albumin (LABTEST), C-Reactive Protein (LABTEST), and hemoglobin (LABTEST).

### Protein expression of cytokines (TGFβ1, TGFβ2, TGβ3, TNFα, IL6, IL2, IL5, IL8, IL13, IL17) employing Luminex® technology

Approximately 100–200 mg of the subcutaneous AT from each sample were homogenized in 300 μL of ice-cold extraction protein buffer (10 mM Tris base, 0.01 mM EDTA, 0.1 mM Sodium Chloride and 1% Triton X-100) to which a protease inhibitor cocktail was added (1 tablet/50 ml extraction buffer) (Roche Diagnostics). Protein extraction from isolated adipocytes was performed in the same way as described above, and the adipocytes were isolated following the adapted protocol from Rodbell [36]. The homogenate was then centrifuged at 18,000 *g* for 40 min at 4 °C and the fatty layer, discarded. The supernatant was stored in aliquots at -80 °C. Multi-species TGFβ 3 plex magnetic bead panel Milliplex® MAP (TGFBMAG-64 K-03, Merck Millipore) was adopted to detect TGFβ1, TGFβ2, and TGFβ3. Before the assay, all samples were centrifuged to remove debris. For the TGFβ assay, all samples were acidified with 1 N HCL, after centrifugation. The Human Cytokine Magnetic Bead Panel Milliplex® MAP (HCYTMAG-60 K-PX29, Merck Millipore) assay was employed to with plasma samples to detect the following cytokines: TNFα, TNFβ, IL1β, IL2, IL5, IL13, IL15, and IL17. All working standards were submitted to serial dilutions, after the reconstitution of stock solutions. MagPlex® beads were mixed with samples, and incubated by 2 h on a plate shaker at room temperature. After the incubation with detection antibody for 1 h, the streptavidin-phycoerythrin was employed to detect the fluorescent reporter intensity from each microsphere that was linked to the sample. The Luminex 200™ instrument with an xMAP® technology system and xPONENT® acquisition software were employed to capture component detection. MILLIPLEX® Analyst 5.1 software was adopted integrating data acquisition and analysis.

### Gene expression

Total RNA was extracted with Trizol® reagent (Invitrogen, Carlsbad, CA) following the manufacturer’s recommendations. The concentration of total RNA was assessed with the Biotek® SynergyH1 spectrophotometer. The cDNA was obtained in a thermocycler (Applied Biosystems Veriti® Thermal Cycler/US.) by reverse transcription (RT) using 1 μg of total RNA from each sample per reaction, with random primers for High-Capacity cDNA Reverse Transcription Kits (Invitrogen no. 4375575), in a final volume of 20 μl. Reverse transcription was performed in a single cycle, which included: i) 10 min at 25 °C; ii) 120 min at 37 °C; iii) 5 s at 85 °C; and, iv) cooling at 4 °C. The obtained cDNA samples were stored at -20 °C until the experiment. PCR was performed using FAST SYBR Green PCR Master Mix (Applied Biosystems, Foster City, CA) in QuantStudio™ 12 K Flex real-time PCR (Applied Biosystems) with primers designed based on GenBank’s database. The mRNA expression of the following genes was determined: RPL27 (NM 000988.3), Foward: CCG AAA TGG GCA AGT TCA T, Reverse: CCA TCA TCA ATG TTC TTC ACG A; SMAD3 (NM_005902.3) Forward: CTA CAG CCA TTC CAT CCC CG, Reverse: AGG TTT GGA GAA CCT GCG TC; SMAD4 (NM_005359.5), Foward: CAC AAG TCA GCC TGC CAG TAT, Reverse: AAT CCA TTC TGC TGC TGT CC; S100A4 (NM_002961.2), Forward: TCT TGG TTT GAT CCT GAC TGCT, Reverse: GTC CTT TCC CCC AAG AAG CTG. Target gene expression was normalized to the reference gene RPL27. The data were analyzed with the 2-ΔΔCT method [2] and expressed as ratio of gene expression normalized to the control sample.

### Histological analysis

Subcutaneous adipose tissue samples (50–100 mg) were obtained after carefully excision with a scalpel blade and fixed in 4% paraformaldehyde (w/v) in pH 4.7 Phosphate buffer, followed by dehydration in absolute ethanol, diaphanization in xylene and embedding in paraffin (Paraplast X-TRA, SIGMA-ALDRICH). The 5 μm sections were mounted onto slides (Starfrost® Knittel Glass). Deparaffinized and hydrated sections were stained with hematoxylin and counterstained with eosin (*n* = 05 for all study groups) and photographed under light microscopy (Olympus).

### Immunohistochemistry

Deparaffinized sections (5 μm) of subcutaneous adipose tissue were kept at the incubator 37 °C overnight, before the immunohistochemistry protocol was performed. Subsequently, the slides were hydrated with 0.05 M PBS, at room temperature. Antigen retrieval was carried out in 10 mM of Citrate buffer (pH 6.0) or Tris-EDTA buffer (pH 9.0) at 95 °C for 20 min. The activity of endogenous peroxidase was blocked in methanol with 0.3% H_2_O_2_, under light protection. All reactions were performed employing Histostain-Plus, IHC kit, HRP broad spectrum® (Life Technologies), following the manufacturer’s instructions. After adding the blocking substrate included the kit, sections were incubated overnight (4 °C) with primary antibodies: Anti-Collagen I rabbit monoclonal antibody [EPR7785] (1:100, Abcam), Anti-Collagen type III rabbit polyclonal antibody (1:100, Rockland Immunochemicals Inc), Anti-Collagen type VI rabbit polyclonal antibody (1:40, Millipore), Anti-Human Fibronectin mouse monoclonal antibody (1:50, Millipore), αSMA (1:100, Abcam), TGFβ1 (V) rabbit polyclonal antibody (1:50, Santa Cruz Biotechnology) and Smad4 (B-8) mouse monoclonal antibody (1:100, Santa Cruz Biotechnology) in a humidified chamber. Negative controls were obtained omitting the primary antibody. Peroxidase activity was revealed with ImmPACT™ DAB substrate (Vector Laboratories, Burlingame, CA USA) and tissue sections were counterstained with Mayer’s hematoxylin (*n* = 05 for all study groups).

In each sample, the immunolabeled tissue was observed under a light microscope (Olympus). Representative images were acquired using Image ProPlus v.5.2 software (Media Cybernetics, Bethesda, MD, USA).

### Immunofluorescence

After the previously described antigen retrieval procedure and quenched endogenous peroxidase activity, sections of subcutaneous adipose tissue were washed with 0.05 M PBS. The blockage of nonspecific sites was performed with normal goat serum, 10% (Reference: 50197Z Life Technologies, Thermo Scientific, USA) added to 1% albumin in 0.1 M PBS containing 0.2% Triton X- 100 for 1 h, at room temperature. The antibody Smad3 (86 F7) Rabbit mAb (1:50, Reference: # 3122, Cell Signaling) was incubated in a humidified chamber, overnight. After removal of the antibody, the sections were washed with 0.1 M PBS/0.2% Triton X-100 for 5 min. The secondary antibody goat anti-rabbit Alexa 546 (Thermo Scientific, USA) was added for 1 h, protected from light. The slides were mounted using ProLong® Montant Gold Antifade with DAPI (Molecular Probes, Life Technologies, USA).

### Statistical analysis

Data were expressed as the median [1st, quartile; 3rd, quartile]. The difference between the groups was assessed by Kruskal-Wallis followed by Dunn post-test, as data presented a non-parametric distribution. Data for group classification and characterization were expressed as mean ± standard error. In this case, one-way analysis of variance with Tukey post-test was adopted. Differences were considered as statistically significant for *P* < 0.05. The statistical data analysis was carried out with the statistical package software GraphPad PRISM (GraphPad Prism version 5.00 for Windows, GraphPad Software, San Diego California USA, www.graphpad.com”). All statistical procedures were performed with the assistance of the Statistics Sector of the Institute of Biomedical Sciences, USP, under the supervision of Ms. Rosana Prisco.

## Results

### General and clinical characteristics of study groups

The clinical characteristics of patients distributed in the study groups are shown in Table 1. For all groups, similar height and age were found. Current and previous weights for 6–12 months (before inclusion of patients in the study), as reported by patients, did not present significant differences among the groups. As expected, the cancer cachexia group (CC) exhibited a pronounced weight loss with 14% body mass reduction (−8.0 [−12.50;−7.0]), while the weight-stable cancer group (WSC) showed body mass variation less than 4.2%, and no changes were observed for the control group (C). Similar results for BMI index were found for all groups.

In order to assess the biochemical values of the parameters employed to determine the patients clinical condition, we examined the serum levels of C-Reactive Protein, albumin, hemoglobin and the plasma levels of interleukin 6 (IL-6) and TNF alpha (TNFα). The C-Reactive Protein values were increased in CC (4.95-fold and 3.05-fold as compared with C and WSC groups, respectively). Hemoglobin and albumin were significantly reduced in CC, in comparison to the control. Despite the consensus recommendation of albumin levels being around 3.2 g/dL, for cachexia diagnosis, CC showed a variation from 2.79 to 4.58 g/dL. In addition, the plasma levels of IL6 (pg/ml) were elevated in cachexia (4.10 pg/ml), whereas, detection in the control (0.00 [0.00; 0.84] pg/ml) and WSC (0.62 [0.00; 1.77] pg/ml) were low. TNFα levels were also enhanced in cachexia (*p* < 0.02), but only compared with the control group. The results show higher systemic inflammation in cachectic patients represented by higher plasma levels of CRP, IL6 and TNFα.

Other plasma pro-inflammatory cytokines were assessed, as shown in Table 1: interleukin (IL) 2(IL2), IL5, IL8, IL3, IL17. All of which have been linked to the development of fibrosis in several experimental models and diseases [29, 31, 37–39]. There was no detection of IL2 values in the plasma of any of the groups. For IL13 and IL17, the levels were not statistically different among the groups. The enhanced expression of IL5 has been associated with elevated levels of IL13 or IL17 [37–40]. Despite these cytokines not presenting any difference induced by cachexia or cancer, when compared with the control group, the levels of IL5 were found to be increased in CC (*p* < 0.02). CC showed higher values of IL8, (55-fold), (28.91 pg/ml), compared with the control (0.52 pg/ml), and with WSC (4.79 pg/ml). Thus, the differences of inflammatory and pro-inflammatory cytokines were more pronounced in the cachectic patients and can be interpreted as indicative of local inflammation.

### Extracellular matrix proteins are overexpressed in the subcutaneous adipose tissue of cancer cachexia

**Fig. 1:**
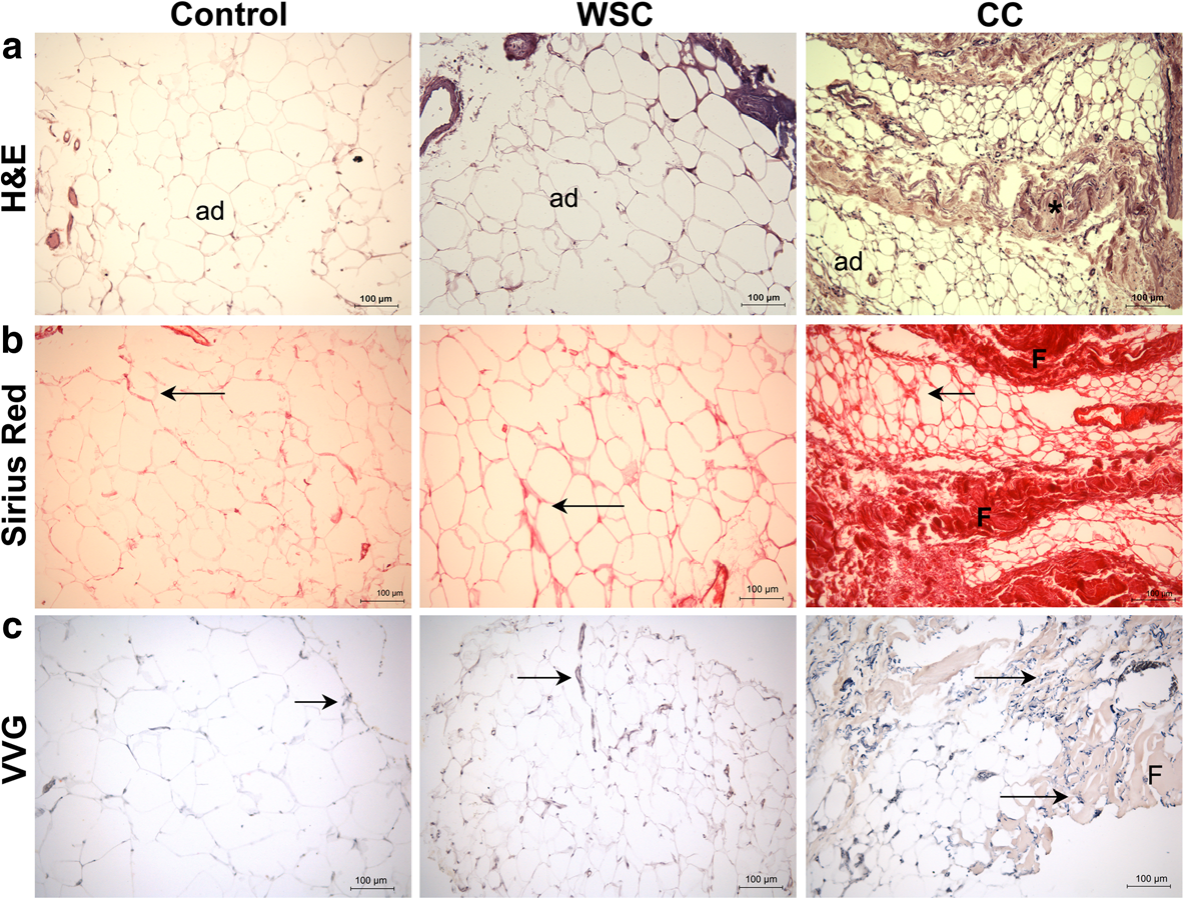
Adipose tissue remodeling is triggered during cancer cachexia. **a** Haematoxilin and eosin sections of subcutaneous adipose tissue. **b** Collagen detection by Picro Sirius Red staining. **c** Localization of elastic fibers performed with Verhoeff’s Van Gieson staining. Photomicrographs of the representative images from each group: Control (*n* = 5), Weight-stable cancer (WSC; *n* = 5), Cancer cachexia (CC; *n* = 5). Ad- Illustrates preserved adipocytes. Arrows indicate intense labeling. F- Indicates areas with excessive ECM deposition (fibrotic areas)

Consistent with our previous findings [35], morphological analysis under light microscopy carried out in haematoxylin and eosin stained sections showed deep modifications in the subcutaneous adipose tissue, exclusively in CC, with differences in the shape of adipocytes and augmented interstitial space (Fig. 1a). The presence of areas with collagen deposits was also more intense in CC compared with control and WSC (Fig. 1b). Along with collagen deposition, we observed an accumulation of mature elastic fibers only in CC (Fig. 1c).

**Fig. 2:**
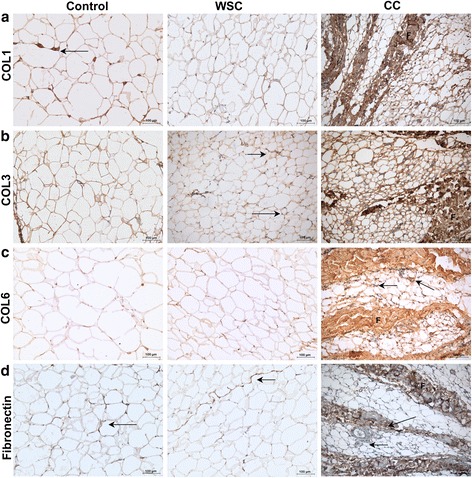
Cachexia induces overexpression of collagen type I, III, VI and fibronectin in the adipose tissue. **a**-**d** Sections of human subcutaneous adipose tissue were immunostained with (**a**) COL1A1, (**b**) COL3A1, (**c**) COL6A1, and (**d**) Fibronectin (FN1). Control (*n* = 5), Weight-stable cancer (WSC; *n* = 5), Cancer cachexia (CC; *n* = 5). Note the positive labeling for all groups, with stronger intensity in CC. Immunostaining for COL6A1 in Control, WSC and CC samples was found around the adipocytes, whereas, in CC, positive staining in several fibrotic areas were observed (F). Ad- Illustrates preserved adipocytes. Blue staining (Mayer’s Haematoxylin) represents a non-reactive nucleous

To characterize the presence of fibrosis in the subcutaneous AT, we investigated by immunohistochemistry, the presence of different types of collagen: type I (COL1), type III (COL3) and type VI (COL6), and of fibronectin (FN). As shown in Fig. 2a, b, c and d respectively, we found similar labeling for COL1, COL3, COL6 and FN for WSC and for the control group. COL6 expression was more evident surrounding adipocytes in all groups than that of COL1 and COL3. The differences were even more pronounced when staining for COL1, COL3, COL6 and FN in the AT of the CC group. In fact, we detected the presence of fibrotic areas in CC, with an accumulation of all the studied collagens along with increased fibronectin expression. In the sections labeled for FN, we observed deposition of fibronectin exclusively in the fibrotic areas.

The overexpression of ECM proteins shown by our results illustrates not only AT remodeling, but the emergence of fibrosis in the AT, due to cancer cachexia.

### Myofibroblasts contributing to fibrosis of adipose tissue in cancer cachexia

**Fig. 3:**
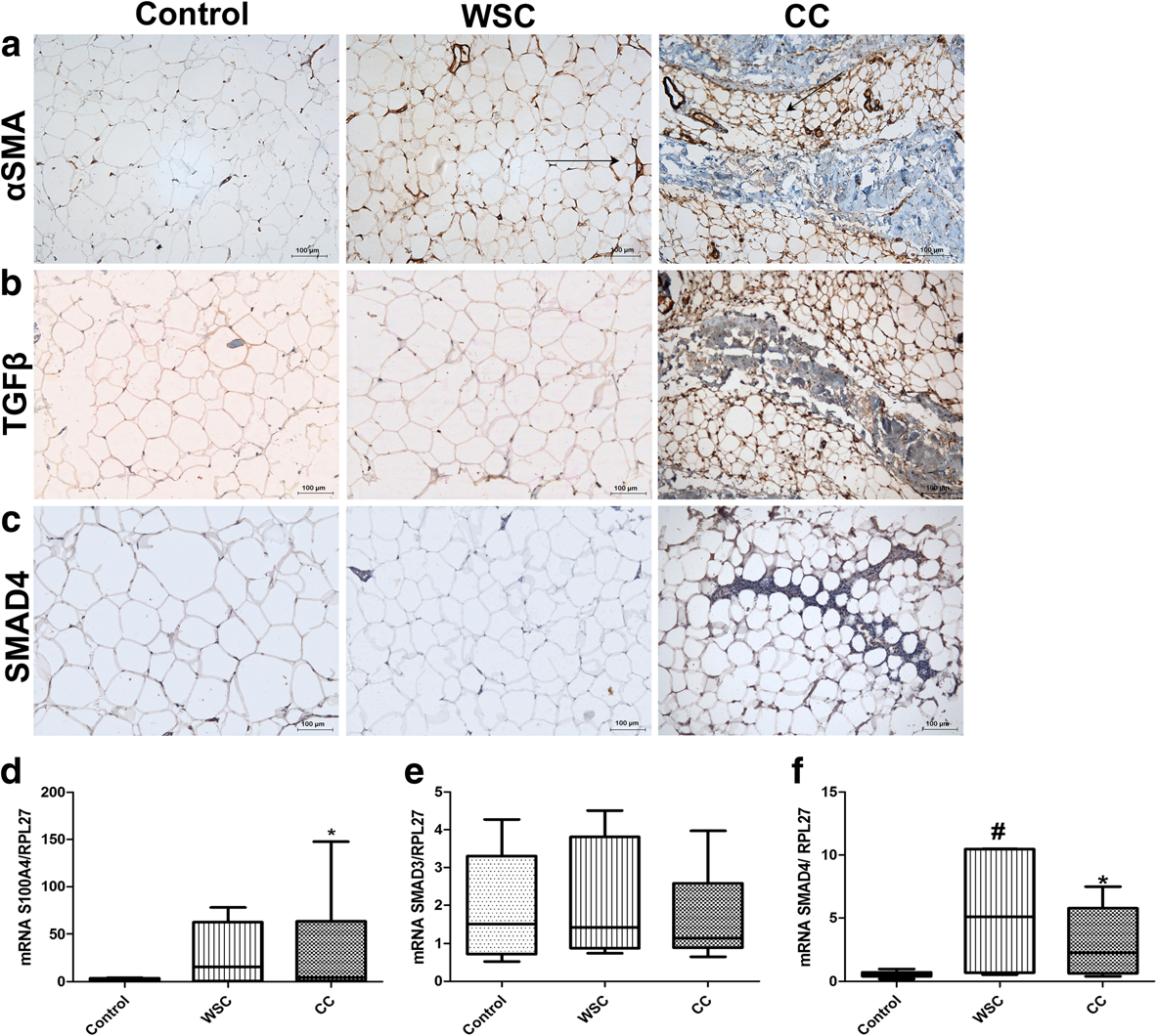
TGFβ and Myofibroblast presence contributes to adipose tissue fibrosis in cancer cachexia. **a** Immunohistochemistry for myofibroblasts with αSMA antibody. Note that almost immunoreactivity in WSC is in vessel walls, whereas CC shows positive cells among adipocytes and in (F) fibrotic areas. **b**-**c** TGFβ immunohistochemical analysis in subcutaneous AT illustrates its possible role in inducing cachexia-associated fibrosis, whereas (**c**) Smad4 shows activation of this pathway. Counterstaining (*blue*) with Mayer’s Haematoxylin was performed. The groups were identified as Control (*n* = 5), Weight-stable cancer (WSC; *n* = 5), Cancer cachexia (CC; *n* = 5). Fibrotic areas are indicated (F). Arrows indicate positive labeling. **d**-**f** qPCR analysis of fibroblast marker (**d**) FSP1 (S100A4) (control, *n* = 13; WSC *n* = 6; CC *n* = 12), (**e**) Smad3 (control, *n* = 9; WSC *n* = 6; CC *n* = 7), and (**f**) Smad4 (control, *n* = 11; WSC *n* = 7; CC *n* = 12). Data presented as median and 1st and 3st quartile. *p < 0.05 CC vs control; # WSC vs control

In order to assess the contribution of fibroblasts with an activated phenotype, myofibroblasts, to the excessive deposition of ECM components in cancer cachexia, we measured α-smooth muscle actin (αSMA) expression. Our results demonstrate (Fig. 3a), as expected, higher expression of αSMA, restricted to vessels walls in sections of WSC when compared to the control group and CC. Interestingly, the presence of myofibroblasts was often found in AT of cachectic patients, specially surrounding adipocytes. The gene expression of the fibroblast-specific protein (FSP1) (also called S100A4) was similarly evaluated. As shown in Fig. 3d, there was a 20-fold increase of FSP1 mRNA levels in CC, compared to controls (*p* < 0.05). Thus, this confirms the presence of activated myofibroblasts with enhanced ECM proteins synthesis due to cachexia.

### TGFβ pathway up-regulated by cachexia

The TGFβ signaling pathway can directly induce myofibroblasts to produce ECM components. To assess the role of TGFβ in ECM remodeling in adipose tissue during cachexia, we detected TGFβ and SMADs proteins expression by different approaches. Immunohistochemistry analysis under light microscopy revealed that there was marked TGFβ1 expression in representative sections from CC group in relation to C and WSC (Fig. 3a). We observed TGFβ1 labeling surrounding each adipocyte, and between the fibrotic areas in the AT from cachectic patients.

**Fig. 4:**
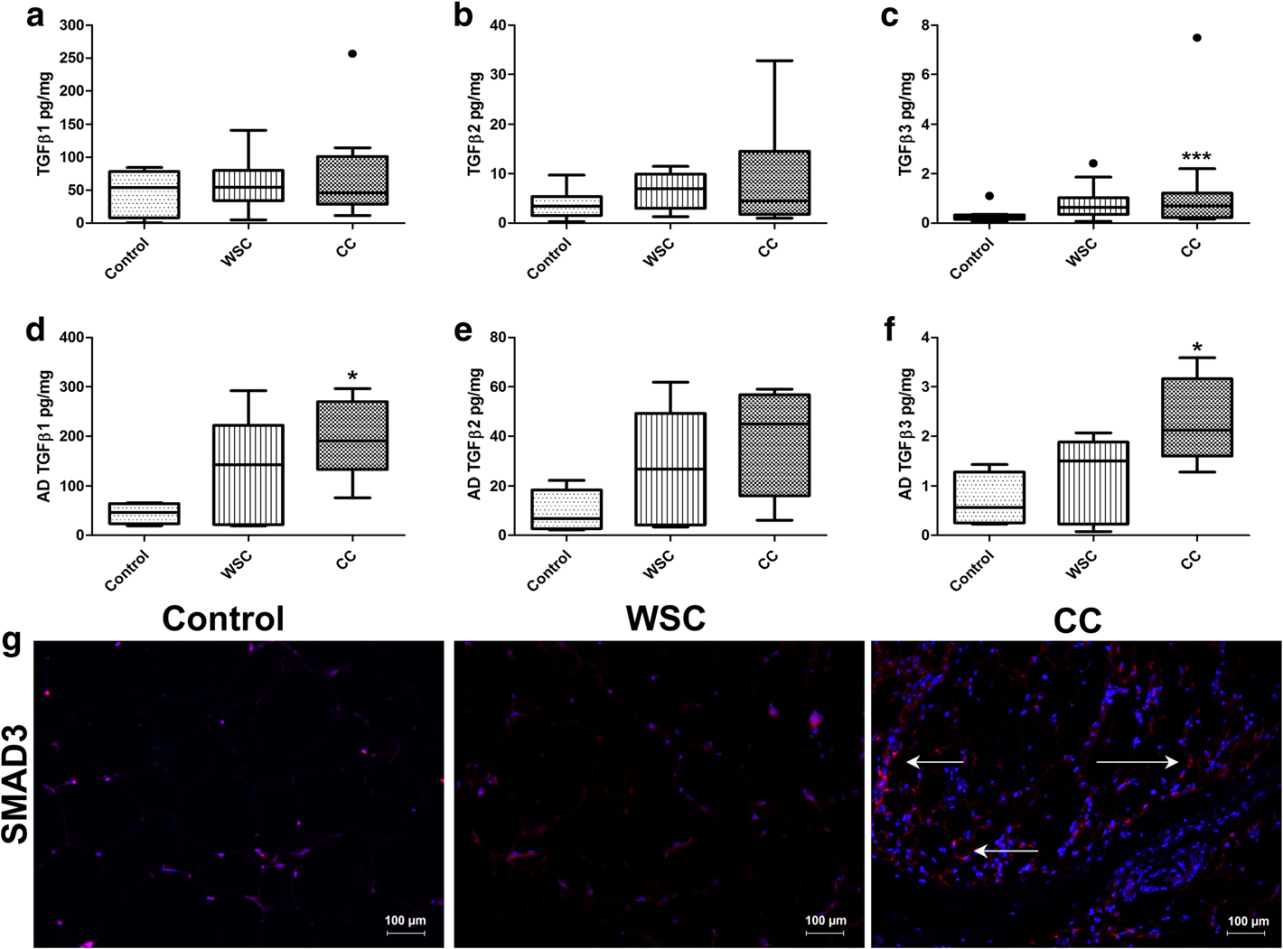
TGFβ pathway is activated in the fibrotic subcutaneous AT in cancer cachexia. **a**-**c** Multiplex assay analysis of activated (**a**) TGFβ1, (**b**) TGFβ2, and (**c**) TGFβ3 in whole subcutaneous AT. Control (*n* = 14), Weight-stable cancer (WSC) (*n* = 12), Cancer cachexia (CC) (*n* = 14). **d**-**f** Adipocytes also contribute for the enhanced observed TGFβ levels, as shown by Multiplex analysis of (**a**) TGFβ1, (**b**) TGFβ2, and (**c**) TGFβ3 from isolated adipocytes of subcutaneous AT. Control (*n* = 5), Weight-stable cancer (WSC) (*n* = 5), Cancer cachexia (CC) (*n* = 5). Data presented as median and 1st and 3st quartile. *p < 0.05, CC vs control; ***p < 0.003 CC vs control. **g** Immunofluorescence staining in subcutaneous AT for Smad3 (red) and DAPI (blue) for nuclei, which showed higher density for Smad3 labeling in cancer cachexia. Control (*n* = 5), Weight-stable cancer (WSC; *n* = 5), Cancer cachexia (CC; *n* = 5)

Smads are proteins which act as downstream signals of the TGFβ pathway. Smad4 interacts with Smad2 and Smad3, forming a complex which rapidly translocates into the nucleus and binds to DNA [26, 41–43]. Firstly, we detected immunolabeling of Smad4 (Fig. 3c) in the AT which was solely present in CC, especially within the fibrotic areas. We analyzed the mRNA levels of Smad3 and Smad4 in the AT (Fig. 3e-f). No differences for gene expression of Smad3 were found, whereas there were increased mRNA levels for Smad4 in WSC and CC groups, both in comparison to the control (*p* < 0.003). The immunofluorescence of Smad3 (Fig. 4g) also demonstrated a more evident expression in CC compared with WSC and the control.

To further explore these results, we quantified the content of the activated isoforms TGFβ1, TGFβ2 and TGFβ3 by Multiplex assay. We failed to find a statistical difference for TGFβ1 levels among the groups (*p* = 0.6055), as shown in Fig. 4a. There were no alterations for isoform β2 in the subcutaneous AT (Fig. 4b). Our results show an increased concentration of TGFβ3 in CC, compared with the controls (*p* = 0.03). In adipocytes isolated from the subcutaneous adipose tissue, (results presented in Fig. 4d-f) an enhanced protein expression of TGFβ1 and TGFβ3 in cachectic patients in relation to the control group (*p* < 0.05) was found. There was a rise of 4-fold and 3.4-fold for TGFβ1 and TGFβ3 concentrations, respectively, in the adipocytes of CC. These results demonstrate that there is a contribution of adipocytes to fibrosis in AT.

Taken together, our findings indicate that there is an augmented activation of the TGFβ pathway in the AT of cachectic patients, revealing an active role of this pathway for disrupted biology tissue and consequent fibrosis.

## Discussion

Cachexia is a wasting disease which affects approximately nine million people worldwide [44]. Cancer cachexia is characterized by progressive body weight reduction due to the loss of skeletal muscle and adipose tissue often associated with systemic inflammation. Current knowledge has shown that adipose tissue depletion in cancer cachexia results from body composition alterations concerning impaired lipid storage capacity and increased lipolysis ratio [13]. We previously showed that the AT suffers morphological modifications which reflects in the reduction of adipocyte size and ECM remodeling in cancer cachexia [35].

Our present findings demonstrate that ECM remodeling of AT in cachexia results not only in augmented collagen fiber content, but also, in excessive elastic fibers and fibronectin deposition. The presence of fibrosis was associated with an increased number of myofibroblasts and an activated TGFβ/SMAD pathway in the subcutaneous AT of gastrointestinal cancer cachectic patients.

In animal models for cancer cachexia (MAC16 tumor bearing-mice) shrunken adipocytes and increased collagen-fibril content in WAT were reported [9]. AT is embedded in a thick ECM surrounding each adipocyte. Nakajima et al. [45] described the presence of type I–VI collagens, laminin and fibronectin in differentiated bovine pre-adipocytes (BIP). Our data show that collagen deposition is accompanied by excessive synthesis of mature elastic fibers, compromising the microenvironment of adipose tissue due to cachexia. We also explored each collagen isoform modified by cachexia. There was strong labeling for collagen type I (COL1) and III (COL3) in the AT from cachectic patients, without differences for control and weight-stable cancer patients, indicating the contribution of both types of collagens for disruption of adipose tissue. Studies with obese subjects reported the presence of fibrous bands in the AT, accessed by Sirius Red staining, whereas in lean individuals, the content of collagen was not changed. In addition, increased amounts of collagen VI (COL6) were found in obese rather than in lean subjects [19].

The expression of COL6 seems to be more specific for adipose tissue since it exhibited a higher expression among different depots of fat, in comparison with liver, muscle, heart, and pancreas, in normal conditions, whereas, the absence of COL6 resulted in an uninhibited expansion of adipocytes [20]. High levels of COL6 in the AT are directly associated with inflammation and fibrosis in obese individuals and in animal models. In obese subjects, immunohistochemistry analysis revealed a stronger label for COL6 and increased mRNA levels of subunit alpha 1 (COL6A1), and both were highly correlated with CTGF and TGFβ levels [46]. The subunit alpha 3 of collagen VI (COL6A3) was also found increased in diabetic obese animals, as well as in obese subjects, with a positive correlation between increased visceral adipose tissue mass and inflammation, independent of BMI; whereas the expression in the subcutaneous AT remained unchanged [20, 47]. The complete assembly of all three chain subunits; α1 (COL1A1), α2 (COL6A2) and α3 (COL6A3) is necessary for stable formation and functionality of COL6 [48]. In this study, we demonstrate the positive presence of COL6A1 in the subcutaneous AT of non-cachectic patients. However, there was a higher expression of COL6A1 in the samples of cachectic patients, accompanied by the presence of intensely labeled bundles in CC.

We report that all of the studied collagen types (COL1A1, COL3A1, and COL6A1) in the AT, to be modulated by cancer cachexia. Fibronectin (FN) has been reported to be found in colocalization with collagen fibers from secretory fibroblasts, while FN absence impairs collagen assembly [49]. The observed changes in collagen deposition dysregulated by cachexia are, in fact, accompanied by augmented FN in the AT of CC. The fibrotic areas were also positively labeled for FN in CC. In studies with obese patients, a strong staining for FN was found in fibrotic regions, the presence of which was increased in the serial sections of AT [50]. Our data demonstrate which ECM proteins induce alterations in the morphology of fat tissue promoting the emergence of fibrosis, in association with cancer cachexia.

Cancer cachexia is orchestrated, in part, by a network of inflammatory factors that are activated for signaling pathways from each tissue joining tumor-derived factors that are the result of malignant inflammation [6]. Consistent with international consensus for cachexia diagnosis, our results demonstrate systemic inflammation in cancer cachectic vs. non-cachectic patients, with increased circulating CRP, IL6, TNFα, IL8, and IL5 levels. A previous study reported that high plasma concentrations of IL8 in patients with advanced lung cancer (stage IV), were correlated with cachexia [51]. On the other hand, increased circulating levels of IL8 are also reported as markers in patients with pulmonary fibrosis [52]. The expression of IL13 and IL17 cytokines has an important role in tissue remodeling and fibrosis. IL13 acts directly to induce collagen production in fibroblasts, thus stimulating proinflammatory mediators, macrophages and dendritic cells [37, 53]. IL17 produced by Th17 cells contributes to the innate and adaptive immune responses, as well to the perpetuation of inflammation with neutrophilia, which in turn, induces tissue damage and fibrosis [38]. In addition, IL17 expression is correlated in the pathogenesis of pulmonary fibrosis, myocardial fibrosis and hepatic fibrosis [38]. Despite this, our study did not presented high levels of IL13 or IL17, whereas, the IL5 expression was significantly enhanced in the plasma from cachectic patients. In the liver fibrosis, the increased levels of IL5 lead to progress of tissue damage by regulating IL13 production and macrophage activation [39]. During states of chronic inflammation, the presence of infiltrating macrophages can augment the myofibroblast activation, tissue damage and fibrosis [23, 53, 54].

Myofibroblasts are contractile cells overexpressing α-SMA and specialize in synthesizing ECM proteins in wound healing responses [23, 29]. α-SMA expression has a critical role in cell motility and contractility during tissue repair, but is also associated to the induction of a fibrotic state [34]. A 3 T3 cell lineage of fibroblasts transfected with α-SMA resulted in cell contractility, which is doubled in lung fibroblasts expressing high levels of α-SMA via TGFβ1 pathway [30]. Mechanical stress caused by excessive contractility leads to an aberrant wound-healing response and fibrosis [33]. In epithelial-mesenchymal cell transition (EMT), which occurs in pulmonary fibrosis, is seen the concomitant increased amounts of mRNA levels of α-SMA, FSP1 and vimentin [55]. According with this, our study showed enhanced immunolabeling of α-SMA in the subcutaneous AT of cancer cachectic group, as well as increased mRNA expression of FSP1. Taken together, these results reveal that changes in collagen and fibronectin expression are associated with increased amounts of activated fibroblasts in cachexia.

TGFβ, a multifunctional cytokine, presents anti-inflammatory and pro-fibrotic activity. This dual role leads to different responses in multiples biological pathways depending of context for their action [23, 56]. TGFβ expression is robustly correlated with the development of fibrosis in the liver, lung, kidney, skin and in cardiac tissues under pathological conditions [23]. In WAT, increased expression of TGFβ has also been reported in obese mice (ob/ob) [57, 58] and in human subjects [59, 60]. In the AT from obese patients and/or rodents, fibrotic areas are frequent [9, 61]. Interestingly, TGFβ1 was increased in subcutaneous AT from cancer cachectic patients, and it was up-regulated in the whole tissue samples, as well as in the isolated adipocytes. The TGFβ3 isoform was elevated solely in the adipocytes, demonstrating the contribution of these cells to the fibrotic state of the AT. The three isoforms: TGFβ1, TGFβ2, and TGFβ3, most expressed in mammals, induce different biological effects. The TGFβ1 isoform seems to play a more important role in wound response and fibrosis [28].

The Smads are a family of transcriptional activators triggered in response to TGFβ. Heterocomplexes formed by R-Smad (Smad2/Smad3) with Smad4 are required to bind DNA regulating gene expression [24]. In knockout models for Smad3, accelerated wound-healing response with low inflammation is demonstrated [24]. TGFβ signaling pathway inhibition has been shown to reduce the development of fibrosis in experimental models [23]. On the other hand, in obesity, expansion of adipocytes is associated with tissue remodeling and fibrosis. The expression of Smad3 is also enhanced in ob/ob mice in fat depots and Smad3 inhibition in vitro results in deficient adipocyte differentiation [57].

Our main interest was to investigate the relationship among adipose tissue fibrosis in cachectic cancer patients and activation of the TGFβ canonical pathway. TGFβ is a cytokine able to activate a wide array of additional cellular functions through non-canonical pathways, such as Rho-like GTPase signaling [62]. In cultured adipocytes, an enhanced Rho-Rho-kinase signaling results in activation of NF-κB and TNFα [63]. In vivo, the inflammatory changes induced by Rho-Rho-kinase signaling, contribute to deregulated adipocytokine expression and recruitment of inflammatory cells to the adipose tissue, which in turn, exacerbates the inflammatory scenario described in obesity [64]. The activation of Rho-kinase by Rho, in addition to Smads signaling triggered by TGFβ in the adipose tissue of cachectic patients could lead to the aggravation of inflammation and, consequently, to fibrosis, but, a detailed study investigating the multiple mechanisms activated by Rho-Rho-kinase during cachexia is necessary.

The subcutaneous AT of cachectic patients show activation of the TGFβ pathway via Smads. We report herein, an increased expression of TGFβ occurs in parallel with Smad3 and 4, thus, demonstrating there was a cytoplasmatic translocation of the Smad complex to the nucleus. Considering this, we suggest that TGFβ/Smad signaling contributes to remodeling, and consequently to fibrosis of AT in cancer cachexia. We speculate that the fibrotic response of AT during cachexia may be triggered by systemic inflammation, concomitant with the activation myofibroblasts contributing to ECM deposition and tissue contractility, through TGFβ/Smad signaling.

## Conclusions

Our study shows the presence of fibrosis in the subcutaneous adipose tissue due to cachexia. In this process, the TGFβ canonic pathway seems to play an important role. The re-arrangement of adipose tissue in cachexia includes elastic fibers, collagens -especially type I, III, VI- and fibronectin. Additionally, the presence of myofibroblasts surrounding adipocytes along with the increased pro-inflammatory cytokine content, indicate severe tissue damage. Extracellular matrix components are crucial for adipose tissue biology. In fact, uncontrolled deposition of collagens, and of other ECM proteins, can prevent the expansion of adipocytes thus leading to the failure of treatments aiming at recovering adipose tissue wasting in cachectic patients.

## Abbreviations

AT: Adipose tissue
CC: Cancer cachexia
ECM: Extracellular matrix
FN: Fibronectin
FSP1: Fibroblast-specific protein
IL: Interleukin
TGFβ: Transforming growth factor beta
WAT: White adipose tissue
WSC: Weight-stable cancer
αSMA: Alpha-smooth muscle actin
